# Exclusion of pregnancy in dialysis patients: diagnostic performance of human chorionic gonadotropin

**DOI:** 10.1186/s12882-020-01729-5

**Published:** 2020-02-28

**Authors:** Natalja Haninger-Vacariu, Harald Herkner, Matthias Lorenz, Marcus Säemann, Andreas Vychytil, Martin Jansen, Rodrig Marculescu, Reinhard Kramar, Gere Sunder-Plassmann, Alice Schmidt

**Affiliations:** 1 Division of Nephrology and Dialysis, Department of Medicine III, Medical University of Vienna, Währingergürtel 18-20, 1090 Vienna, Austria; 2 Department of Emergency Medicine, Medical University of Vienna, 1090 Vienna, Austria; 3 Dialysis Centre Vienna, 1220 Vienna, Austria; 4 Department of Medicine VI, Wilhelminenspital, 1160 Vienna, Austria; 5 Sigmund Freud Private University, Medical School, 1020 Vienna, Austria; 6 Division of Gastroenterology and Nephrology, Department of Medicine I, Hospital St. John of God, 1020 Vienna, Austria; 7 Department of Laboratory Medicine, Medical University of Vienna, 1090 Vienna, Austria; 8 Austrian Dialysis and Transplant Registry, 4532 Rohr im Kremstal, Austria

**Keywords:** Anesthesia, Chronic kidney disease, Diagnostic accuracy, Dialysis, Fertility, Menopause, Menstrual cycle, Human chorionic gonadotropin, Follicle stimulating hormone, Anti-Müllerian hormone, Immunosuppressant, Kidney transplantation, Pregnancy, Surgery

## Abstract

**Background:**

A positive pregnancy test in acute or chronically ill patients has implications for the use of potentially mutagenic or teratogenic products in urgent medical therapies such as the use of chemotherapies or therapies with immunosuppressants, for anesthesia, and for time-sensitive indications like urgent surgery or organ Transplantation.

Despite a lack of evidence, it is currently believed that human chorionic gonadotropin serum concentrations are always elevated in female dialysis patients even without pregnancy. It is also believed that human chorionic gonadotropin cannot be used to confirm or exclude pregnancy.

**Methods:**

Human chorionic gonadotropin was examined in female dialysis patients (18–50 years of age), and was classified as positive above 5 mlU/ml. In addition, fertility status was determined. For an enhanced index test, the cut-off of 5 mIU/ml was used for potentially fertile patients and 14 mIU/ml for infertile patients to calculate diagnostic test accuracy. The ideal cut-off for human chorionic gonadotropin was estimated using Liu’s method with bootstrapped 95% confidence intervals. Predictors of human chorionic gonadotropin increase were analyzed using multivariable linear regression.

**Results:**

Among 71 women, two (2.8%) were pregnant, 46 (64.8%) potentially fertile, and 23 (32.4%) infertile. We observed human chorionic gonadotropin concentrations > 5 mIU/ml in 10 patients, which had a sensitivity of 100% (95% confidence interval: 100 to 100), a specificity of 86% (95% confidence interval: 77 to 94), a positive predictive value of 17% (95% confidence interval: 8 to 25) and a negative predictive value of 100% (95% confidence interval: 100 to 100) for the diagnosis of pregnancy. Using a cut-off > 14 mIU/ml for infertile patients or the exclusion of infertile patients increased specificity to 93% or 98%, respectively. The ideal cut-off was 25 mIU/ml (95% confidence interval: 17 to 33). Pregnancy and potential fertility, but not age, were independent predictors of human chorionic gonadotropin.

**Conclusion:**

Human chorionic gonadotropin is elevated > 5mIU/ml in 14.5% of non-pregnant dialysis patients of child-bearing age. In potentially fertile women, this cut-off can be used to exclude pregnancy. In case of an unknown fertility status, the ideal human chorionic gonadotropin cut-off was 25 mIU/ml.

## Background

Traditional teaching suggests that surgery and general anesthesia in pregnancy should be postponed until after delivery to avoid unnecessary risks to the fetus [1]. However, reluctance to operate during pregnancy might become a self-fulfilling prophecy in which delay of surgery contributes to adverse perinatal outcomes traditionally attributed to surgery itself [2].

In this context, it is important to mention that kidney transplantation in pregnant dialysis patients can be associated with poor fetal outcome [3]. Thus, physicians should rule out pregnancy in kidney transplant candidates of childbearing age, especially in case of mycophenolic acid (MPA) use during initial immunosuppression. Although MPA significantly reduces incidence of acute rejection in general, MPA use in pregnancy is associated with an increased risk of miscarriage and congenital defects [4]. Therefore, the Food and Drug Administration mandates pregnancy testing immediately before and eight days following initiation of immunosuppressive therapy with MPA as part of a risk evaluation and mitigation strategy [4].

The diagnosis or exclusion of early pregnancy among dialysis patients is thought to be demanding because it is generally believed that human chorionic gonadotropin (hCG) serum concentrations can be elevated in dialysis patients even without pregnancy [5]. However, during the last four decades only three case reports and four small case series described 11 post-menopausal dialysis patients and nine dialysis patients of reproductive age showing elevated hCG serum concentrations in a range suggestive of gestational weeks 3 to 5 [6–12]. Despite these results, dialysis patients were neither pregnant nor presented with malignancy. In addition to hCG testing, transvaginal ultrasonography represents a useful tool for pregnancy investigation. Although, transvaginal ultrasonography can only identify a gestational sac at 5 weeks of gestational age with a diameter > 5 mm [13]. Thus, anesthesiologists and transplant practitioners are faced with a diagnostic window of several weeks when pregnancy in dialysis patients or kidney transplant candidates with elevated hCG serum concentrations cannot safely be excluded.

We aimed to examine the diagnostic performance of hCG serum concentrations for the exclusion or diagnosis of pregnancy in an Austrian sample of female dialysis patients less than 50 years of age. The results may inform decisions on diagnostic thresholds and use of pregnancy testing in this patient population.

## Methods

Between May 2016 and December 2017, consecutive female dialysis patients of childbearing age (defined as 18 to 50 years of age) from four dialysis units in Vienna, Austria (Dialysis Centre Vienna, Hospital St. John of God, Medical University of Vienna, Wilhelminenspital) were prospectively included in this study.

Eligible patients were identified using the Austrian Dialysis and Transplant Registry, which has provided nearly 100% coverage of patients on chronic renal replacement therapies in Austria since 1965. Patient history and clinical data including current pregnancies were obtained by baseline and follow-up interviews, and chart review.

The institutional review boards (IRB) of all four participating centers approved the study (unique IRB identifier for Medical University of Vienna: 750/2016; Dialysis Centre Vienna and Hospital St. John of God: 27–2-17; Wilhelminenspital: EK 17–058-VK). All participants gave written informed consent. Investigations were in accordance with the Declaration of Helsinki.

Blood samples were collected before dialysis and serum concentrations of hCG, follicle stimulating hormone (FSH), luteinizing hormone (LH), and anti-Müllerian hormone (AMH) were measured using the Roche cobas® 8000 modular analyzer, immunoassay module (e 602, Roche Diagnostics International Ltd., Rotkreuz, Switzerland) by electrochemiluminescence immunoassay (ECLIA). At first, the AMH Gen II ELISA© 2015 (Beckman Coulter, Inc. 250 S. Kraemer Blvd., Brea, CA 92821 U.S.A.), an enzymatically amplified two-site immunoassay, was used for measurement of AMH.

Reference intervals provided by the manufacturers of the laboratory tests were used to classify serum hormone concentrations as low, normal or elevated (Table S1). All analyses were performed in one International Organization for Standardization 15,189 accredited clinical laboratory of the Department of Laboratory Medicine at the Medical University of Vienna

The definition of true menopause in dialysis patients is not unequivocal because, in the absence of a menstrual cycle, women can regain fertility with hormone therapy, intensified dialysis and after transplantation [14]. We used the gynecological history (including menstrual cycle: yes, irregular, no; oophorectomy: yes or no) and hormonal profile to classify patients as pregnant, potentially fertile or infertile [14]. The potentially fertile patients included women with ovulatory or suspected functional anovulatory cycles representing patients without menopause or with functional menopause. The infertile women had pre- or true menopause. According to the Stages of Reproductive Aging Workshop (STRAW) criteria, an elevated FSH and low AMH concentration were used to ascertain infertility [15]. In dialysis patients, an elevation of FSH concentrations also indicates true menopause [14]. Serum AMH concentrations were found to be similar in dialysis patients and healthy controls [16, 17] and thus we used low AMH concentrations to ascertain infertility in selected cases. We also described LH serum concentrations, which are usually elevated in case of menopausal transition, infertility or in dialysis patients [18, 19].

Categorized data are presented as absolute count and relative frequency. Continuous data are presented as mean ± standard deviation, if they are approximately normally distributed, or as a median value with 25–75% interquartile ranges. We used standard methodology to calculate summary statistics for diagnostic tests, including sensitivity, specificity, positive and negative predictive values with 95% confidence intervals. The reference test was pregnancy, the index test was hCG. A cut-off of 5mlU/ml was used to classify patients as positive for elevated levels of hCG [20]. For an enhanced index test, we classified hCG as positive above a cut off at 5 mlU/ml for potentially fertile patients and 14 mlU/ml for infertile patients [21]. A potential stratum effect of potential fertility on the summary statistics for diagnostic tests was investigated by use of stratification. In addition, the ideal cut-off for hCG was estimated using the Liu method, which maximizes the product of sensitivity and specificity in the ROC space [22]. The estimated cut-off value was determined with a bootstrapped 95% confidence interval.

Predictors of hCG increase were analyzed using multivariable linear regression. Human chorionic gonadotropin was the outcome on the log-transformed scale; age (years), pregnancy (yes versus no), and potential fertility (yes versus no) were used as covariables. MS Excel and Stata 14 for Mac were used for data management and analyses. In general, a two-tailed *p*-value less than 0.05 was considered statistically significant.

This study was conducted and the manuscript prepared according to the Standards for Reporting Diagnostic Accuracy (STARD) statement and the Quality Assessment of Diagnostic Accuracy Studies 2 (QUADAS-2) [23, 24].

## Results

**Fig. 1:**
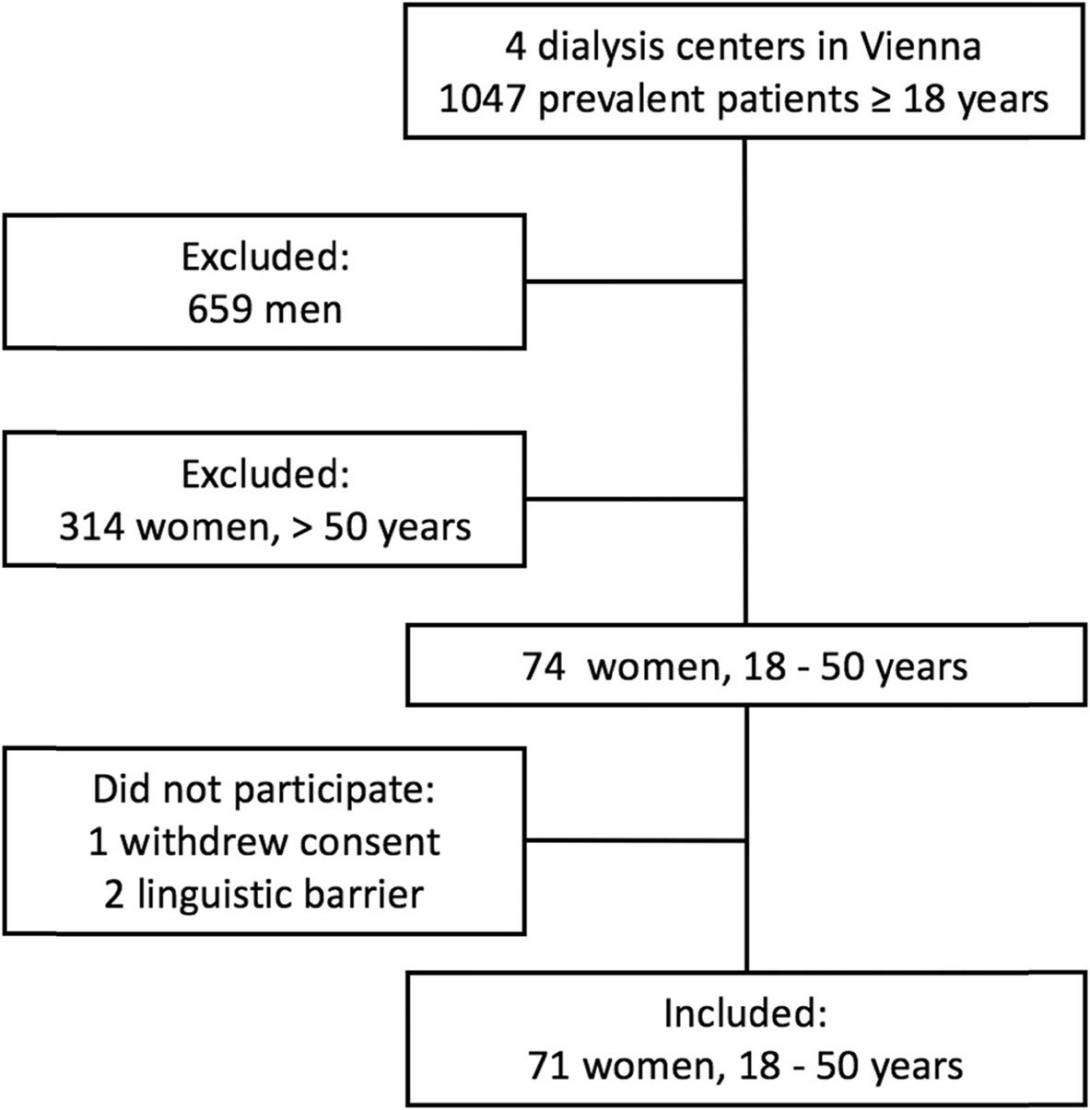
Patient disposition

During the 19-month study period, 71 consecutive female dialysis patients aged 18–50 years were enrolled from four dialysis centers in Vienna. This study sample represents roughly 32% of the Austrian dialysis population of this sex and age category (point prevalence of dialysis patients > 18 years of age in Austria as of December 31st 2016: 4584; point prevalence of female dialysis patients aged 18–50 as of December 31st 2016: 221). The detailed assembly of the study cohort is given in Fig. 1. Overall rate of patient participation in the four study centers was about 96%.

**Fig. 2:**
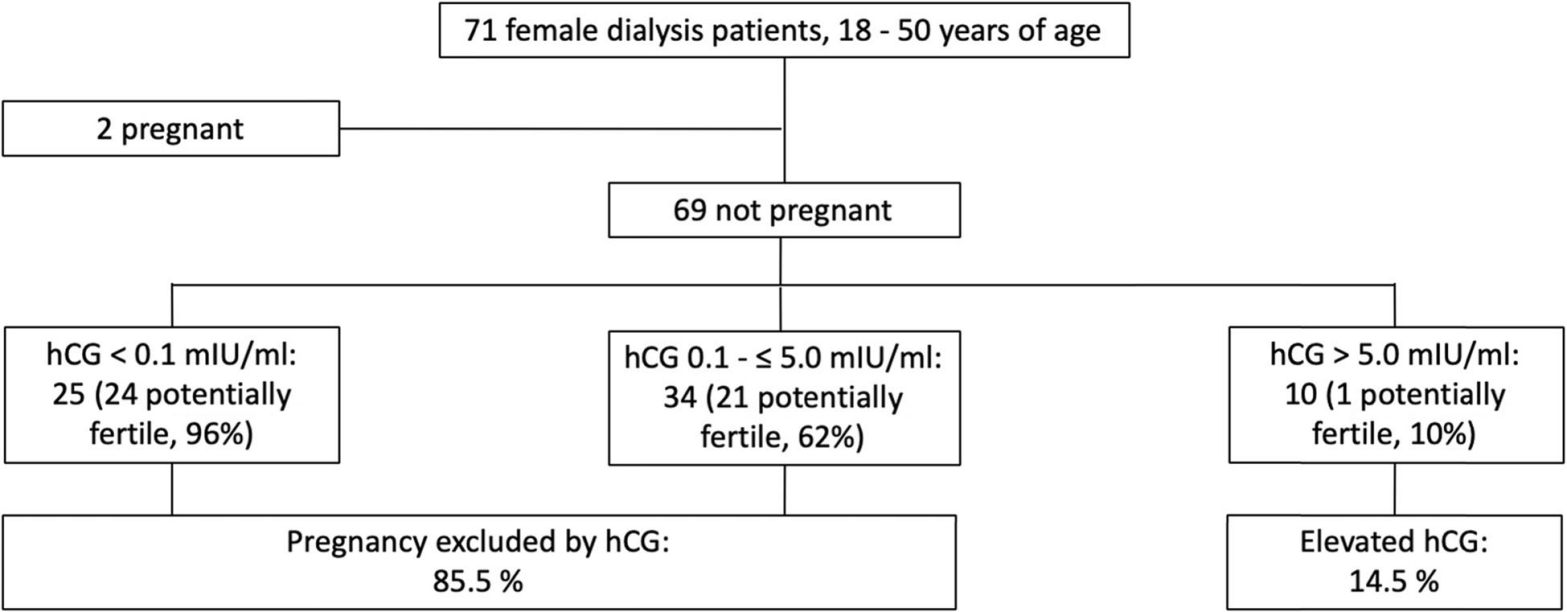
Distribution of hCG serum concentrations among 71 female dialysis patients. The higher the hCG serum concentration, the lower the proportion of potentially fertile individuals in the different hCG serum concentration categories. hCG, human chorionic gonadotropin

**Fig. 3:**
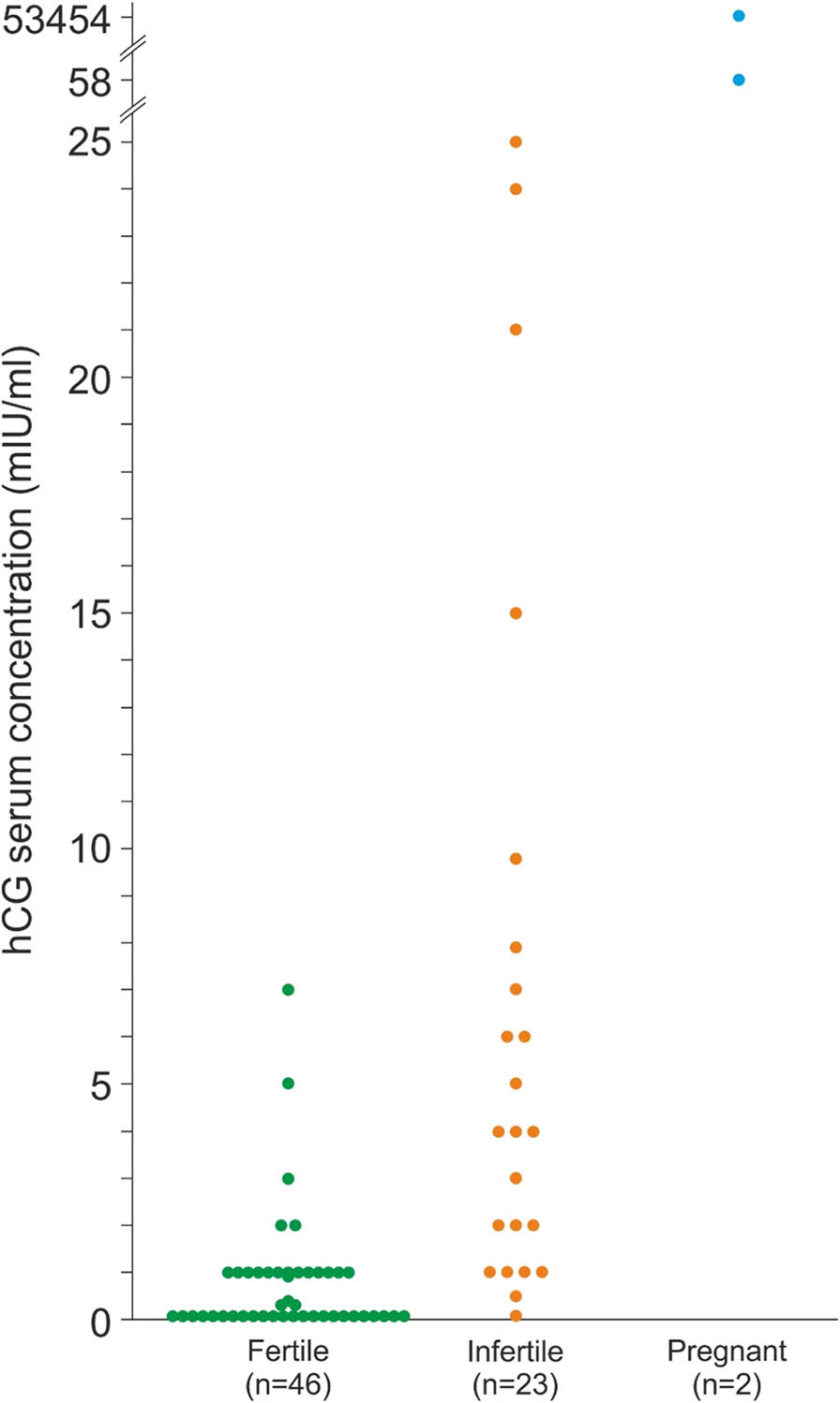
Individual hCG serum concentrations of 71 potentially fertile, infertile, and pregnant dialysis patients. hCG, human chorionic gonadotropin

Among the 71 enrolled women, two (2.8%) were pregnant, one did not know at the time of blood sampling and both presented with distinct elevated hCG serum concentrations; both pregnancies were recognized after initiation of the study. At the time of blood sampling for this study 34% of the patients were waitlisted for kidney transplantation with a Eurotransplant waitlist status “transplantable” and 17% with a Eurotransplant waitlist status “not transplantable”. Demographic data, information on primary kidney disease and end-stage renal disease vintage of the entire study cohort is given in Table 1. Table 2 depicts the gynecological and obstetric history of all patients. 46 women were classified as potentially fertile and 23 as infertile (Table 3). The median serum concentrations of hCG, LH, FSH, and AMH of 69 non-pregnant women and of menstrual cycle and menopausal status subgroups are indicated in Table 4. 25 cases presented with an undetectable serum hCG of < 0.1 mlU/ml, 34 cases with levels up to 5 mlU/ml, and we observed concentrations > 5 mlU/ml, potentially indicating pregnancy, in 10 patients (14.5% of the non-pregnant cohort; Fig. 2). Follow-up, including personal interviews, excluded malignancies associated with elevated hCG concentrations, pregnancies or miscarriages in all 10 patients. Individual hCG serum concentrations according to pregnancy and fertility status are given in Fig. 3. Table 5 shows demographic details and serum hormone concentrations of 12 women with hCG serum concentrations > 5 mlU/ml, including two pregnant cases. One of the 10 non-pregnant women with an hCG serum concentration > 5 mlU/ml was considered potentially fertile, the other nine infertile. Clinical details, individual serum hormone concentrations and fertility status of 59 patients with hCG serum concentrations ≤5 mlU/ml are indicated in Table S2.

**Table 1:** Demographic details of 71 female dialysis patients

Characteristics	*n* = 71
Age, years	43 (32–47)
Age ≤ 40 years	30 (42%)
Age, 40–50 years	41 (58%)
BMI, kg/m^2^	23.1 (20.6–27.7)
Race, black, white, Asian	1 (1%)/65 (92%)/5 (7%)
Renal disease	
Diabetes	4 (6%)
Glomerulonephritis	9 (13%)
Secondary glomerulonephritis/vasculitis	11 (15%)
Interstitial nephritis/pyelonephritis	7 (10%)
Cystic/hereditary/congenital diseases	14 (20%)
Neoplasms/tumors	3 (4%)
Complication after transplantation	2 (3%)
Miscellaneous conditions	2 (3%)
Unknown	18 (25%)
Total ESRD vintage, years	2.3 (0.8–11.2)
Total dialysis time, years	2.1 (0.7–4.4)
Number of patients with	
1, 2, 3, 4 previous kidney transplants	17 (24%), 2 (3%), 2 (3%), 1 (1%)
Duration of current dialysis, years	1.6 (0.4–2.9)
Current hemodialysis/peritoneal dialysis	60 (85%), 11 (15%)

Data expressed either as median (IQR) for continuous variables or as count and percentage for categorical variables

*BMI* body mass index, *ESRD* end-stage renal disease

**Table 2:** Gynecological and obstetric history of 71 female dialysis patients

Characteristics	*n* = 71
Age of menarche, years	13 (12–14)
Total number of pregnancies^a^	147
Number of pregnancies per patient	1 (0–3)
Number of patients with	
unknown, no, 1, 2, 3, 4, 5, 7, 10, 11 pregnancies^a^	1/28/8/8/10/3/6//4/1/1
Total number of live births	90
Number of live births per patient	1 (0–2)
Number of patients with	
unknown, no, 1, 2, 3, 4, 5, 8 live births	1/33/12/11/8/1/4/1
Current regular menstrual cycle (including 2 pregnant women)	31 (44%)
Current irregular menstrual cycle	13 (18%)
Current amenorrhea	25 (35%)
Current menstrual cycle unknown	2 (3%)
No/functional/pre/true/unknown menopause	36/11/8/15/1
History of oophorectomy	5 (7%)
Prior therapy with cyclophosphamide	7 (10%)
Potentially fertile patients	46 (65%)

Data expressed either as median (IQR) for continuous variables or as count and percentage for categorical variables

^a^ Two pregnancies during dialysis included in the present study, two other pregnancies during dialysis before the present study

**Table 3:** Patient categories, fertility status and menstrual cycle subgroups of 71 dialysis patients of reproductive age

	Potentially fertile (*n* = 46) No or functional menopause^a^	Infertile (*n* = 23) Pre- or true menopause^b^	Pregnant (*n* = 2) No menopause^c^
Regular menstrual cycle (*n* = 29)	Ovulatory or functional anovulatory cycle (*n* = 24) (low AMH: 3, AMH unknown: 1)	Based on history and/or elevated FSH (*n* = 5) (low AMH: 4)	Regular menstrual cycle before pregnancy (n = 2)
Irregular menstrual cycle (*n* = 13)	Ovulatory or functional anovulatory cycle (*n* = 11) (low AMH: 1)	Based on history and/or elevated FSH (*n* = 2) (low AMH: 2)	–
Amenorrhea (*n* = 25)	Functional menopause (*n* = 10) (low AMH: 3)	Based on history and/or elevated FSH (*n* = 15) (low AMH: 12)	–
Unknown menstrual cycle (*n* = 2)	Ovulatory or functional anovulatory cycle (*n* = 1) (low AMH: 0)	Based on history and/or elevated FSH (*n* = 1) (low AMH: 1)	–

*FSH* follicle stimulating hormone, *AMH* anti-Müllerian hormone

^a^, low AMH: 7 of 46 (15%); ^b^, low AMH: 19 of 23 (83%); ^c^, low AMH: 0 of 2 (0%)

**Table 4:** Serum hormone concentrations of non-pregnant dialysis patients and of subgroups according to menstrual cycle status and menopausal status

Hormone			Serum concentration			
	Non-pregnant	Menstrual cycle (*n* = 67)^a^			Menopause (*n* = 69)	
		Regular	Irregular	Amenorrhea	No or functional	Pre- or true
(manufacturer and method)	(*n* = 69)	(*n* = 29)	(*n* = 13)	(*n* = 25)	(*n* = 46)	(*n* = 23)
hCG (Roche, ECLIA), mlU/ml	1.0 (< 0.1–2.0)	1.0 (0.1–1.0).	0.1 (0.1–1.0)	1.0 (0.5–6.0)	0.1 (0.1–1.0)	4.0 (1.5–7.45)
LH (Roche, ECLIA), mlU/ml	9.45 (3.9–33.2)	10.15 (3.98–27.6)^b^	7.7 (4.2–11.6)	10.4 (3.6–95.5)	7.1 (3.6–15.7)^b^	82.3 (4.4–120.8)
FSH (Roche, ECLIA), mlU/ml	5.40 (3.90–22.7)	5.0 (3.55–9.75)^b^	4.4 (3.9–6.0)	21.7 (4.2–80.0)	4.6 (3.6–6.3)^b^	58.3 (18.5–117)
AMH (Beckman-Coulter, ELISA; *n* = 33), ng/ml	0.16 (< 0.08–3.2)	0.66 (0.1–3.44)	4.2 (0.86–6.3)	0.08 (< 0.08–0.33)	0.73 (0.31–5.46)	0.08 (< 0.08- < 0.08)
n	33	14	6	12	21	12
AMH (Roche, ECLIA; *n* = 35), ng/ml	0.19 (0.01–0.8)^b^	0.36-(0.15–1.62)^b^	0.37 (0.2–0.96)	0.01 (0.01–0.04)	0.38 (0.18–1.71)^b^	0.01 (0.01–0.03)
n	35^b^	14^b^	7	13	24^b^	11

*hCG* human chorionic gonadotropin, *LH* luteinizing hormone, *FSH* follicle stimulating hormone, *AMH* anti-Müllerian hormone, *ECLIA* electrochemiluminescence immunoassay, *ELISA* enzyme linked immunosorbent assay

Data expressed as median (IQR); ^a^ unknown menstrual cycle status in two patients; ^b^ 1 missing

**Table 5:** Clinical details of 10 non-pregnant dialysis patients with hCG concentrations > 5 mIU/ml and two pregnant dialysis patients

Patient No.	Age categories (years)	BMI categories (kg/m^2^)	PD or HD	ESRD vintage (years)	Pregnancies (count)	Live births (count)	Current menstrual cycle (y/n/u/i)	Cycle day of blood sampling	hCG (mIU/ml)	LH (mIU/ml)	FSH (mIU/ml)	AMH (ng/ml)	No, functional, pre-, or true menopause (N, F, P, T)	Comment
Non-pregnant dialysis patients														
1	45.0–50.0	18.5–24.9	HD	23.1	5	3	n	–	6.00	83.4	142	< 0.08^a^	T	Infertile, elevated FSH^b^
2	45.0–50.0	18.5–24.9	HD	2.30	11	8	n	–	6.00	187	172	< 0.08^a^	T	Infertile, elevated FSH
3	45.0–50.0	18.5–24.9	HD	0.13	1	1	n	–	21.0	125	58.3	< 0.08^a^	T	Infertile, elevated FSH
4	45.0–50.0	< 18.5	HD	1.53	0	0	n	–	24.0	< 0.10	1.00	< 0.08^a^	T	Infertile^c^
5	45.0–50.0	< 18.5	HD	2.27	0	0	n	–	25.0	> 200	> 200	< 0.08^a^	T	Infertile, elevated FSH^b^
6	20.0–24.9	18.5–24.9	HD	19.6	0	0	r	u	7.00	1.30	3.10	3.89^a^	N	Potentially fertile, high prolactin, hCG decreased at follow-up
7	20.0–24.9	18.5–24.9	HD	0.97	0	0	r	1	9.80	4.60	65.2	< 0.08^a^	P	Infertile, elevated FSH
8	45.0–50.0	25.0–29.9	HD	25.1	1	1	n	–	7.90	116	105.8	0.01	T	Infertile, elevated FSH
9	45.0–50.0	< 18.5	HD	1.10	0	0	n	–	7.00	< 0.10	0.90	0.10	T	Infertile^c^
10	45.0–50.0	≥30	HD	1.63	1	1	n	–	15.0	82.3	69.7	0.01	T	Infertile, elevated FSH^b^
Pregnant dialysis patients														
11	25.0–29.9	≥30	HD	0.16	4	3	–	–	53454^d^	< 0.10	< 0.10	3.64	N	Fertile, delivery of a healthy child
12	45.0–50.0	25.0–29.9	HD	2.08	10	2	–	–	58.0^e^	13.7	17.1	0.05	N	Fertile, missed abortion (1st trimester)

*No*. number, *BMI* body mass index, *HD* hemodialysis, *PD* peritoneal dialysis, *ESRD* end-stage renal disease, *hCG* human chorionic gonadotropin, *LH* luteinizing hormone, *FSH* follicle stimulating hormone, *AMH* anti-Müllerian hormone, *y* yes, *n* no, *u* unknown, *i* irregular, *N* no, *F* functional, *P* pre, *T* true

^a^, AMH measured by ELISA (Beckman-Coulter); ^b^, received a kidney transplant during follow-up and remained infertile with elevated serum hCG concentration; ^c^, low LH & FSH related to hypogonadotropic hypogonadism caused by anorexia; ^d^, week 16 + 4 of gestation; ^e^, week 4 + 1 of gestation

An hCG serum concentration > 5 mlU/ml had a sensitivity of 100% (95% CI: 100 to 100), specificity of 86% (95% CI: 77 to 94), positive predictive value of 17% (95% CI: 8 to 25) and negative predictive value of 100% (95% CI: 100 to 100) for the diagnosis of pregnancy. Using an hCG cut-off of > 14 mlU/ml for infertile patients, sensitivity and negative predictive value did not change, the specificity increased to 93% (95% CI: 87 to 99) and positive predictive value was 29% (95% CI: 18 to 39). Within the stratum of potentially fertile patients, specificity was 98% (95% CI: 94 to 100) with a positive predictive value of 67% (95% CI: 53 to 80). Sensitivity and negative predictive value remained unchanged. Cross tabulations of pregnancy as the reference standard and hCG as an index test are shown in Table S3. Overall, the ideal hCG cut-off for our patient population was 25.0 mlU/ml (95% CI: 17 to 33).

Pregnancy (coefficient: 8.7 (if pregnant); 95% CI: 6.6 to 10.7; *p* < 0.001) and potential fertility (coefficient: − 2.7 (if potentially fertile); 95% CI: − 3.5 to − 1.8; p < 0.001) were independent predictors of hCG levels. In contrast, age had no effect on hCG serum concentrations (coefficient − 0.03 (per year of age); 95% CI: − 0.08 to 0.01; *p* = 0.13).

## Discussion

We provide evidence that serum concentrations of hCG are elevated in only 2.2% of potentially fertile female dialysis patients of reproductive age, in contrast to 39% of infertile patients. This finding has implications for the exclusion of pregnancy in surgery settings and anesthesia in general, for time-sensitive indications such as deceased donor kidney transplantation, and for the use of MPA as a part of primary immunosuppression.

Fertility is diminished in dialysis patients with a pregnancy incidence between < 1–7% [5, 25–29]. Menopause occurs in these women 4.5 years earlier than in healthy women and primary ovarian failure, defined as a sign of menopause before the age of 40 years, is frequent with a proportion of 14% in comparison to 0.01% in the general population [30–32]. Profound endocrine abnormalities leading to menstrual and fertility disorders in dialysis patients are likely the result of a defect in hypothalamic regulation of gonadotropin secretion [33] and in the absence of other clinical correlates, serum concentrations of hCG may be elevated in these patients. When they do conceive, advanced renal failure predisposes them to abortion, intrauterine growth restriction and preterm delivery. Intensified dialysis may improve fertility and pregnancy outcomes among dialysis patients, [34] but the rate of successful pregnancies is about four times greater after kidney transplantation (33/1000 female transplant recipients) but still less frequent as compared to the general population (> 100/1000 females) [35, 36]. Notably, two studies showed an increase of the pregnancy rates among dialysis patients in recent years [37–39].

Tacrolimus and MPA are recommended as first line therapy for prevention of kidney allograft rejection [40]. However, MPA use in pregnancy is associated with an increased risk of miscarriage with a rate of 45–49% during the first trimester, and congenital defects, such as external ear malformation, cleft lip and palate, and abnormality of distal limbs, heart, esophagus or kidneys, which occur in 23–27% of cases. In comparison, the miscarriage rate in female solid organ recipients receiving other immunosuppression lies between 12 and 33% and the rate of congenital defects between 4 and 5%, which is comparable to 3% in the general US population [1]. Therefore, the FDA and EMA recommend pregnancy exclusion immediately before starting with MPA therapy, eight days later, and the use of contraceptives during ongoing therapy. Furthermore, the Report by the American Society of Anesthesiologists Task Force on Preanesthesia Evaluation suggested that pregnancy testing may be offered to female patients of childbearing age and for whom the result would alter the patient’s management, because patients may present for anesthesia with early undetected pregnancy [41]. Surgery and anesthesia on a pregnant woman may have significant implications for the fetus and the mother, and it is commonly recommended that all surgery, unless truly emergent, be postponed until after delivery to minimize the risk to the fetus [2]. However, withholding indicated surgery from a pregnant woman as a result of fears of teratogenesis, pregnancy loss, or preterm birth would appear to be unfounded and may significantly contribute to both maternal and neonatal morbidity [3]. Taken together, an undetected pregnancy in a dialysis patient who undergoes kidney transplantation and uses MPA as part of the immunosuppressive therapy poses an unacceptable risk to the fetus [4].

Pregnancy can be diagnosed or excluded by measurement of hCG in urine or serum. It is a heterodimeric glycoprotein hormone composed of an alpha- and a specific beta-subunit. The hCG alpha-subunit is identical to the alpha-subunit of LH, FSH, and thyroid-stimulating hormone (THS) and before release into circulation, the alpha- and beta-subunits are non-covalently bound. Human chorionic gonadotropin is normally secreted by the syncytiotrophoblast of the placenta, but also by trophoblastic and gastrointestinal tumors. The main functions of hCG include the maintenance of progesterone secretion from the corpus luteum until the placenta takes over this function after 6 weeks of gestation; it also stimulates gonadal testosterone secretion of the male fetus. Very small amounts in men and women primarily derive from the anterior pituitary gland [42, 43]. Some 8% of menopausal women present with elevated serum concentrations of hCG of pituitary origin > 5 mlU/ml and a higher cut-off of 14 mlU/ml is recommended for women > 55 years [21].

Today, serum hCG is measured by highly specific 2-site immunometric assays using antibodies specific for the beta-subunit [20]. The urine hCG pregnancy tests detect the free beta-subunit and are less sensitive as compared to serum assays. In dialysis patients, urine tests are not recommended and not possible because of anuria, and serum hCG based pregnancy testing is reported to be unreliable due to a high rate of “false” positive results [9]. However, due to the lack of reliable data, this suggestion is only supported by a few case reports in the literature (summarized in Table S4).

Therefore, hCG serum concentrations were examined with a highly sensitive and specific test in female dialysis patients of reproductive age. Potential fertility and infertility were diagnosed with a detailed medical and gynecological history and measurement of serum concentrations of FSH, AMH and LH. Except for two pregnant patients, 46 out of 69 non-pregnant cases (67%) were classified to be potentially fertile and 23 as infertile. Only one of the non-pregnant, potentially fertile women (2.2%) presented with an hCG serum concentration > 5 mlU/ml (case 6 in Table 5). Nine further patients with elevated hCG > 5 mlU/ml (6 to 25 mlU/ml) were considered infertile, which represented 39% of the infertile patient group. Thus, among female dialysis patients of reproductive age, 14.5% presented with elevated serum hCG, and the vast majority of them were classified as infertile. In contrast to age, potential fertility and pregnancy were independent predictors of hCG serum concentrations.

In regard to diagnostic accuracy, this study showed that the hCG cut-off of > 5 mlU/ml had a specificity of 86% for the diagnosis of pregnancy among the group of female dialysis patients of childbearing age as a whole. Using a higher cut-off of 14 mlU/ml for the subgroup of infertile patients, specificity increased to 93%. For the group of potentially fertile patients alone, specificity improved to 98%. The positive predictive value increased accordingly in all three analyses, whereas sensitivity and the negative predictive value remained at 100%. Thus, for potentially fertile dialysis patients of childbearing age a standard hCG serum concentration cut-off of ≤5 mlU/ml can be used to safely exclude early pregnancy. In case of an unknown fertility status, the ideal cut-off for the diagnosis of pregnancy in our patient population was 25 mlU/ml, which corresponds well to the cut-off suggested by Braunstein et al. [20]. When a patient presents with an elevated hCG serum concentration, causes other than pregnancy or menopause should be considered. There may be malignancies or test interference with heterophilic antibodies, which are frequently encountered in patients with autoimmune diseases [44, 45].

Potential limitations to the study should also be considered, such as the small sample size which diminishes the generalizability of our data. Due to this, the wide confidence intervals reflect random sampling variability. However, given the importance of the outcome in light of the very low frequency of this condition, it appears to be a disproportionate effort to recruit more participants only to gradually increase precision before reporting this observation. Likewise, a confidence interval around the 100% predictive value is a result of no observations rather than a high precision. We cannot exclude the possibility of false negatives with a larger sample size. Moreover, by avoiding a case control study design, it was possible to exclude an important source of selection bias. Blinding of the index test was assured by the analysis of hCG in a department of laboratory medicine. There was no blinding of the reference standard, but we do not deem this a major source of bias for advanced pregnancy. However, this may not be the case for miscarriages. A stratum bias does not seem likely because patients were recruited from several institutions without traceable selection.

## Conclusion

Our data show that highly specific serum hCG tests can be used in potentially fertile female dialysis patients to exclude pregnancy. While waitlisted for kidney transplantation or scheduled for elective surgery and anesthesia, an elevated hCG serum concentration in women of childbearing age should prompt an evaluation for the presence of functional, pre-, or true menopause, and include a gynecological history and determination of FSH and AMH serum concentrations.

In conclusion, the serum concentration of hCG was elevated > 5 mlU/ml in nearly half of infertile dialysis patients of childbearing age. In contrast, for potentially fertile women this cut-off can be used to exclude pregnancy. For the population as a whole, the ideal hCG cut-off was 25 mlU/ml.

## Supplementary information

**Additional file 1: Table S1.** Reference intervals for hCG, FSH, LH, and AMH in females. **Table S2.** Clinical details of 59 female dialysis patients with hCG serum concentrations of ≤5 mIU/ml. **Table S3.** Cross tabulation of pregnancy as the reference standard and hCG as index test. **Table S4.** Studies reporting elevated serum concentrations of hCG in 20 female dialysis patients.

## Data Availability

All data generated or analyzed during this study are included in this published article [and its supplementary information files]. Furthermore, data are available from the corresponding author on reasonable request.

## Abbreviations

AMH: Anti-Müllerian hormone
ECLIA: Electrochemiluminescence immunoassay
FSH: Follicle stimulating hormone
hCG: Human chorionic gonadotropin
IRB: Institutional review boards
LH: Luteinizing hormone
MPA: Mycophenolic acid
QUADAS-2: Quality Assessment of Diagnostic Accuracy Studies 2
STARD: Standards for Reporting Diagnostic Accuracy
STRAW: Stages of Reproductive Aging Workshop
THS: Thyroid-stimulating hormone
