# Supplementary material for: Exclusion of pregnancy in dialysis patients: diagnostic performance of human chorionic gonadotropin
Source: BMC Nephrol. 2020 Feb 28;21:70. doi: 10.1186/s12882-020-01729-5 (PMC7049197; doi:10.1186/s12882-020-01729-5)
Supplement: Supplementary file 1 — Additional file 1: Table S1. Reference intervals for hCG, FSH, LH, and AMH in females. Table S2. Clinical details of 59 female dialysis patients with hCG serum concentrations of ≤5 mIU/ml. Table S3. Cross tabulation of pregnancy as the reference standard and hCG as index test. Table S4. Studies reporting elevated serum concentrations of hCG in 20 female dialysis patients. [file 12882_2020_1729_MOESM1_ESM.doc]

**Supplemental material**

Exclusion of pregnancy in dialysis patients:

diagnostic performance of human chorionic gonadotropin

Natalja Haninger-Vacariu, Harald Herkner, Matthias Lorenz, Marcus Säemann,

Andreas Vychytil, Martin Jansen, Rodrig Marculescu, Reinhard Kramar,

Gere Sunder-Plassmann, Alice Schmidt

**Table of contents** **Page**

**Table S1.** Reference intervals for hCG, FSH, LH, and AMH in females 2

**Table S2.** Clinical details of 59 female dialysis patients with hCG serum concentrations of ≤ 5 mIU/ml 3

**Table S3.** Cross tabulation of pregnancy as the reference standard and hCG as index test 5

**Table S4.** Studies reporting elevated serum concentrations of hCG in 20 female dialysis patients 6

**References**  7

**Table S1**. Reference intervals for hCG, FSH, LH, and AMH in females

**_________________________________________________________________________________**

**Hormone** **Reference interval**

**_________________________________________________________________________________**

**hCG (ECLIA, Roche Diagnostics) mIU/ml (5th to 95th percentile)**

Nonpregnant 0-5.3

Postmenopausal -8.3

Pregnant (gestational week)

3 5.8-71.2

4 9.5-750

5 217-7138

6 158-31795

7 3697-163563

8 32065-149571

9 63803-151410

10 46509-186977

12 27832-210612

14 13950-62530

15 12039-70971

16 9040-56451

17 8175-55868

18 8099-58176

**_________________________________________________________________________________**

**FSH (ECLIA, Roche Diagnostics) mIU/ml (5th to 95th percentile)**

Follicular phase 3.5-12.5

Ovulation phase 4.7-21.5

Luteal phase 1.7-7.7

Postmenopausal 25.8-134.8

**_________________________________________________________________________________**

**LH (ECLIA, Roche Diagnostics) mIU/ml (5th to 95th percentile)**

Follicular phase 2.4-12.6

Ovulation phase 14-95.6

Luteal phase 1-11.4

Postmenopausal 7.7-58.5

**_________________________________________________________________________________**

**AMH** **(ECLIA, Roche Diagnostics) ng/ml (5th to 95th percentile)**

Age (years)

20 - 24 1.22-11.7

25 - 29 0.89-9.85

30 - 34 0.58-8.13

35 - 39 0.15-7.49

40 - 44 0.03-5.47

45 - 50 0.01-2.71

**_________________________________________________________________________________**

**AMH** **(ELISA, Beckman Coulter)* ng/ml**

Age > 18 years <12.6

Day 3 of menstrual cycle <10.6

Postmenopausal <0.08

**_________________________________________________________________________________**

hCG, human chorionic gonadotropin; FSH, follicle stimulating hormone; LH, luteinizing hormone;

AMH, anti-Mullerian hormone; ECLIA, electro chemiluminescence immunoassay; ELISA, enzyme-

linked immunosorbent assay.

* used for study patients No. 1-24 and 37-45.

**Table S2.** Clinical details of 59 female dialysis patients with hCG serum concentrations of ≤ 5 mIU/ml

___________________________________________________________________________________________________________________________________________________________________________________

Patient Age BMI PD ESRD Pregnancies Life Current Cycle hCG LH FSH AMH No, functional, Comment

categories categories or vintage births menstrual day of pre-, or true

HD cycle blood menopause

No. (years) (kg/m²) (years) (count) (count) (y/n/u/i) sampling (mIU/ml) (mIU/ml) (mIU/ml) (ng/ml) (N/F/P/T)

1 25.0-29.9 <18.5 HD 16.4 0 0 n - <0.10 6.80 4.40 8.81* F Potentially fertile

2 20.0-24.9 18.5-24.9 HD 0.88 0 0 i 13 <0.10 7.70 6.00 13.0* N Potentially fertile

3 35.0-39.9 <18.5 HD 27.1 0 0 r 18 <0.10 0.50 2.30 0.68* N Potentially fertile

4 40.0-44.9 ≥30 HD 0.47 2 2 i u 1.00 4.20 2.80 <0.08* P Infertile, unmet desire to have a

third child before dialysis, low AMH

5 35.0-39.9 25.0-29.9 HD 2.51 5 3 r 5 2.00 6.10 3.90 2.08* N Potentially fertile, oral

contraception

6 20.0-24.9 18.5-24.9 HD 8.11 0 0 r 21 1.00 24.7 3.40 17.6* N Potentially fertile

7 25.0-29.9 18.5-24.9 HD 14.3 0 0 r 17 <0.10 7.00 2.20 5.46* N Potentially fertile

8 35.0-39.9 18.5-24.9 HD 24.0 0 0 n - 1.00 10.4 8.70 0.59* F Potentially fertile

9 45.0-50.0 25.0-29.9 HD 2.70 0 0 i u 2.00 24.1 12.4 <0.08* P Infertile, age, low AMH

10 45.0-50.0 18.5-24.9 HD 21.1 2 1 r 19 1.00 11.9 3.30 0.73* N Potentially fertile

11 35.0-39.9 25.0-29.9 HD 10.3 0 0 r 1 1.00 63.7 24.7 <0.08* P Infertile, unmet desire to have a

child before HD, high FSH, low AMH

12 45.0-50.0 <18.5 HD 10.3 0 0 u - 5.00 154 57.2 <0.08* T Infertile, elevated FSH

13 45.0-50.0 18.5-24.9 HD 21.0 5 1 r 20 <0.10 10.5 4.30 0.10* N Potentially fertile

14 45.0-50.0 25.0-29.9 HD 20.3 0 0 r 17 2.00 4.00 6.50 <0.08* P Infertile, unmet desire to have a

child before dialysis, low AMH

15 45.0-50.0 <18.5 HD 22.7 3 0 n - 3.00 130 128 <0.08* T Infertile, elevated FSH

16 25.0-29.9 18.5-24.9 HD 0.29 3 2 n - 1.00 3.60 5.20 <0.08* F Potentially fertile

17 25.0-29.9 ≥30 PD 0.06 0 0 r 10 <0.10 1.70 2.40 0.11* N Potentially fertile

18 45.0-50.0 18.5-24.9 HD 0.06 0 0 r 25 <0.10 29.2 22.0 0.01 N Potentially fertile

19 45.0-50.0 18.5-24.9 HD 0.32 0 0 n - 1.00 <0.10 0.50 0.01 P Infertile, ovarian cancer and

chemotherapy, low AMH

20 40.0-44.9 18.5-24.9 HD 1.45 1 1 r 15 1.00 36.2 6.30 2.06 N Potentially fertile

21 40.0-44.9 18.5-24.9 HD 8.26 0 0 r 23 <0.10 32.2 12.0 0.13 N Potentially fertile

22 45.0-50.0 18.5-24.9 HD 0.04 1 1 r u 1.00 3.40 2.70 1.85 N Potentially fertile

23 25.0-29.9 u HD 0.27 u u u - 1.00 15.7 4.80 1.78 u Potentially fertile

24 40.0-44.9 ≥30 HD 2.23 4 3 r 3 <0.10 3.80 6.70 0.58 N Potentially fertile

25 45.0-50.0 ≥30 HD 1.11 5 4 r 1 <0.10 3.90 3.60 0.38 N Potentially fertile

26 30.0-34.9 18.5-24.9 HD 0.19 0 0 r 12 0.90 27.0 18.4 0.33 N Potentially fertile

27 45.0-50.0 <18.5 HD 0.14 4 3 n - 0.50 4.00 27.0 0.04 T Infertile, elevated FSH

28 35.0-39.9 18.5-24.9 PD 0.44 3 2 r 3 <0.10 9.80 4.40 0.64* N Potentially fertile

29 45.0-50.0 25.0-29.9 PD 0.81 2 1 n - 5.00 3.50 4.20 0.31* F Potentially fertile

30 25.0-29.9 18.5-24.9 PD 12.1 0 0 i u 3.00 16.7 4.40 6.66* N Potentially fertile

31 30.0-34.9 18.5-24.9 PD 5.17 0 0 i 30 <0.10 47.0 5.20 3.20* N Potentially fertile

32 45.0-50.0 ≥30 PD 2.84 0 0 n - 1.00 3.90 4.20 0.40* F Potentially fertile

33 45.0-50.0 ≥30 PD 1.67 0 0 n - 1.00 8.50 6.20 0.16* F Potentially fertile

34 45.0-50.0 25.0-29.9 HD 0.02 7 5 r 19 1.00 25.7 5.40 0.12* N Potentially fertile

35 18.0-19.9 18.5-24.9 PD 1.81 0 0 r 27 2.00 50.2 4.60 17.6* N Potentially fertile

36 40.0-44.9 18.5-24.9 PD 29.9 2 0 i 19 <0.10 7.70 3.90 5.20* N Potentially fertile

37 30.0-34.9 25.0-29.9 HD 0.46 2 2 i 18 <0.10 2.30 1.60 5.81 N Potentially fertile

38 20.0-24.9 <18.5 HD 0.72 0 0 r 4 <0.10 7.10 7.00 2.48 N Potentially fertile

39 40.0-44.9 25.0-29.9 HD 13.9 3 3 n - 1.00 69.3 155 0.01 T Infertile, elevated FSH

40 40.0-44.9 18.5-24.9 HD 5.61 2 2 n - <0.10 <0.10 0.90 0.01 F Potentially fertile

41 35.0-39.9 18.5-24.9 PD 2.91 5 5 i 11 1.00 6.80 3.90 0.37 N Potentially fertile

42 45.0-50.0 25.0-29.9 HD 0.19 3 2 r 20 4.00 >200 190 0.01 P Infertile, elevated FSH

43 40.0-44.9 ≥30 HD 5.30 5 5 n - 4.00 98.7 80.0 0.01 T Infertile, elevated FSH

44 35.0-39.9 25.0-29.9 HD 5.40 2 2 i 50 <0.10 2.00 4.60 1.23 F Potentially fertile

45 45.0-50.0 <18.5 HD 4.00 1 1 n - 4.00 95.5 66.2 0.01 T Infertile, elevated FSH

46 40.0-44.9 25.0-29.9 HD 2.58 1 1 n - <0.10 5.30 21.7 0.07 F Potentially fertile

47 35.0-39.9 18.5-24.9 HD 14.3 1 1 r 1 <0.10 5.20 4.60 0.94 N Potentially fertile

48 40.0-44.9 18.5-24.9 HD 2.27 3 0 n - <0.10 <0.10 0.70 0.01 F Potentially fertile

49 45.0-50.0 ≥30 HD 2.32 3 3 i 26 <0.10 9.10 8.30 0.22 N Potentially fertile

50 45.0-50.0 ≥30 HD 1.75 7 3 r 6 <0.10 2.50 7.30 0.29 N Potentially fertile

51 45.0-50.0 18.5-24.9 HD 0.55 3 2 n - <0.10 27.8 40.8 0.02 T Infertile, elevated FSH

52 45.0-50.0 18.5-24.9 HD 27.7 2 0 r 9 2.00 90.1 54.6 0.05 P Infertile, elevated FSH

53 30.0-34.9 18.5-24.9 HD 2.07 3 2 n - 0.30 19.7 5.40 1.69 F Potentially fertile

54 40.0-44.9 18.5-24.9 HD 0.39 7 5 r 4 0.30 10.8 9.00 0.20 N Potentially fertile

55 30.0-34.9 18.5-24.9 HD 12.5 0 0 i ? <0.10 <0.10 0.30 0.68 N Potentially fertile

56 35.0-39.9 ≥30 HD 1.94 2 1 i 4 <0.10 11.6 4.40 0.12 N Potentially fertile

57 35.0-39.9 18.5-24.9 PD 21.2 0 0 i 7 0.40 4.40 7.20 0.1 N Potentially fertile

58 30.0-34.9 ≥30 HD 1.33 7 2 r 4 1.00 24.6 4.60 3.79 N Potentially fertile

59 30.0-34.9 18.5-24.9 HD 6.49 3 0 r 29 1.00 n.a. n.a. n.a. N Potentially fertile

___________________________________________________________________________________________________________________________________________________________________________________

No., number; BMI, body mass index; HD, hemodialysis; PD, peritoneal dialysis; ESRD, end-stage renal disease; hCG, human chorionic gonadotropin; LH, luteinizing hormone; FSH, follicle stimulating hormone;

AMH, anti-Müllerian hormone; y, yes; n, no; u, unknown; i, irregular; N, no; F, functional; P, pre; T, true; n.a., not available

*, AMH measured by ELISA (Beckman-Coulter).

**Table S3.** Cross tabulation of pregnancy as the reference standard and hCG as an index test

**A**

Pregnant

hCG >5mIU/ml 0 1 Total

0 59 0 59

1 10 2 12

Total 69 2 71

**B**

Potentially fertile: hCG >5mIU/ml Pregnant

Infertile: hCG >14 mIU/ml 0 1 Total

0 64 0 64

1 5 2 7

Total 69 2 71

**C**

Pregnant

Potentially fertile: hCG >5mIU/ml 0 1 Total

0 45 0 45

1 1 2 3

Total 46 2 48

___________________________________________________________________________________________

hCG cut-off of **A**) 5 mIU/ml, **B**) 5 mIU/ml for potentially fertile patients and 14 mIU/ml for infertile patients, and **C**) excluding 23 infertile cases.

hCG, human chorionic gonadotropin.

**Table S4.** Studies reporting elevated serum concentrations of hCG in 20 female dialysis patients

**Reference Cases Age Dialysis vintage Fertility status hCG Comment**

**(years) (years) (serum concentration)**

Schwarz, 1985 9

a, b, c, d, e n.a. n.a. 5 fertile > 10 mIU/ml No information on diagnosis of fertility, *

f, g, h, i n.a. n.a. 4 infertile > 10 mIU/ml No information on diagnosis

of infertility, *

Hubinot, 1986 5

a n.a. n.a. 1 fertile >2 ng/ml No information on diagnosis

of fertility, *

b, c, d, e n.a. n.a. 4 infertile >2 ng/ml No information on diagnosis

of infertility, *

Buckner, 2007 1 35 ~ 7 Irregular m.c. 290 mIU/ml Presence of heterophilic

antibodies possible

Cole, 2007 1 60 n.a. Infertile 32 IU/ml Postmenopausal

Fahy, 2008 1 19 9 Irregular m.c. 33 mIU/ml Patient with lupus

erythematosus,

heterophilic antibodies

possible, premature

ovarian failure possible

DeBacker, 2013 1 29 10 Infertile 26 IU/l Hepatitis C, cryoglobulins,

Presence of heterophilic

antibodies possible

Soni, 2013 2

a 65 7 Infertile 8 IU/l Postmenopausal

b 46 2 Fertile 27 IU/l No information on diagnosis

of fertility

n.a., not available; m.c., menstrual cycle; hCG, human chorionic gonadotropin.

* hCG measured by radioimmunoassay which cross-reacts with luteinizing hormone.

**References**

1. Schwarz A, Post KG, Keller F, Molzahn M. Value of human chorionic gonadotropin measurements in blood as a pregnancy test in women on maintenance hemodialysis. Nephron. 1985;39:341-3.

2. Hubinont C, Doutrelepont JM, Vanherweghem JL, Gervy C, Schwers J. Comparison of human chorionic gonadotropin and pregnancy-specific beta 1-glycoprotein in nonpregnant patients undergoing hemodialysis. Nephron. 1986;43:149-50.

3. Buckner CL, Wilson L, Papadea CN. An unusual cause of elevated serum total beta hCG. Ann Clin Lab Sci. 2007;37:186-91.

4. Cole LA, Sasaki Y, Muller CY. Normal production of human chorionic gonadotropin in menopause. N Engl J Med. 2007;356:1184-6.

5. Fahy BG, Gouzd VA, Atallah JN. Pregnancy tests with end-stage renal disease. J Clin Anesth. 2008;20:609-13.

6. De Backer B, Goffin F, Nisolle M, Minon JM. [Persistent low hCG levels beyond pregnancy: report of two cases and review of the literature]. Ann Biol Clin (Paris). 2013;71:496-502.

7. Soni S, Menon MC, Bhaskaran M, Jhaveri KD, Molmenti E, Muoio V. Elevated human chorionic gonadotropin levels in patients with chronic kidney disease: Case series and review of literature. Indian J Nephrol. 2013;23:424-7.

8. Braunstein GD. False-positive serum human chorionic gonadotropin results: causes, characteristics, and recognition. Am J Obstet Gynecol. 2002;187:217-24.
